# Revisiting Fire Safety Guidelines in CO_2_ Laser Airway Surgery

**DOI:** 10.1002/lary.70078

**Published:** 2025-08-24

**Authors:** Ariel Roitman, Seth H. Dailey, Marcus Wilson, Andrew J. Bowen, Kristopher M. Schroeder, Susan L. Thibeault

**Affiliations:** ^1^ Department of Otolaryngology‐Head and Neck Surgery, School of Medicine and Public Health University of Wisconsin‐Madison Madison Wisconsin USA; ^2^ The Ruth and Bruce Rappaport Faculty of Medicine Technion—Israel Institute of Technology Haifa Israel; ^3^ Department of Anesthesiology University of Wisconsin School of Medicine and Public Health Madison Wisconsin USA

**Keywords:** airway surgery, CO_2_ laser, experimental study, FiO_2_ limit, fire risk, microlaryngoscopy, porcine laryngeal model

## Abstract

**Introduction:**

The potential for airway fire during endoscopic laser surgery is well known, typically addressed by reducing FiO_2_ levels. This study revisits these established practices in CO_2_ laser tubeless airway surgery.

**Materials and Methods:**

Using a cadaveric porcine larynx and lung model, we conducted trials with high‐flow (HFV) and jet ventilation. We varied FiO_2_ levels and laser power settings to record the onset and number of brief, expansive, and blowtorch flames.

**Results:**

Flames were observed in all jet ventilation trials. Multivariable model results showed that increasing laser wattage decreased onset times for expansive/blowtorch flames and increased their frequency (*p* < 0.05). Increased FiO_2_ changes were not significant (*p* > 0.05). HFV trials revealed no expansive flames at 4 W at 100%, 6 W at 70%, and 10 W at 50% FiO_2_. Increasing wattage shortened flame onset and increased frequency across all flames (*p* < 0.01), while increasing FiO_2_ only reduced onset time for brief flames (*p* < 0.05). Jet ventilation led to higher flame incidence and shorter onset times than HFV (*p* < 0.05).

**Conclusion:**

Fire risk during CO_2_ laser surgery in oxygen‐rich environments is influenced more by wattage, rather than FiO_2_ levels. Jet ventilation consistently produced expansive flames, even at lower FiO_2_ levels and wattage, supporting a FiO_2_ limit of 30% for this ventilation technique. Our findings support HFV as a potentially safer option for CO_2_ laser surgery, with safe zones of 4 W at 100%, 6 W at 70%, and 10 W at 50% FiO_2_, provided laser application is under 30 s. Our model's calculation of excess risk from wattage increments can help surgeons assess this fire risk.

## Graphical abstract

This experimental study evaluates fire risk during CO2 laser airway surgery using high‐flow and jet ventilation in a porcine model. Results showed that laser wattage had a greater influence on fire risk than FiO_2_, with jet ventilation consistently producing expansive flames. High‐flow ventilation demonstrated safer parameters, supporting its use with defined FiO_2_‐wattage safety zones.

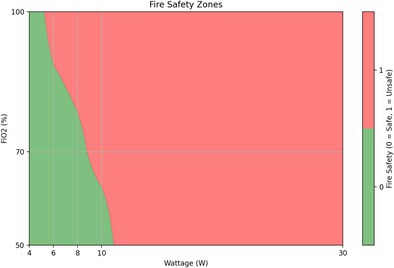

## 1 Introduction

The risk of airway fire during endoscopic laser procedures is well documented, posing the potential for serious injury and catastrophic events [1, 2]. The fire triangle concept, where fuel, an ignition source, and an oxidizer are all required to generate fire, guides our understanding of this risk [3]. Airway surgery teams use precautionary measures to reduce fire risk. For example, teams use special equipment such as laser‐safe endotracheal tubes, low‐ignition risk substances such as soaked pledgets, and low‐ignition risk lasers. Also, selectable features such as laser settings and a lower fraction of inspired oxygen (FiO_2_) are used to control the oxidizer element of the triangle [4, 5, 6, 7, 8, 9, 10]. However, our recent fire risk experiments with the TruBlue laser, a diode laser emitting at 445 nm, suggest that these well‐known precautions may merit re‐examination. While current guidelines recommend reducing FiO_2_ levels to 30% or lower when possible [11, 12, 13], our study provided new insights; we found that it is safe to use the TruBlue laser with a laser‐safe ETT even with 100% oxygen, provided that the cuff is fully inflated and placed 0.3 cm below the operative site. Additionally, we found a low fire risk even in oxygen‐rich environments with high‐flow and jet ventilation conditions [14, 15]. These findings suggest that it is perhaps safer than previously described to use the CO_2_ laser even in high oxygen environments, at least under certain conditions.

With these findings in mind, we used a CO_2_ laser to test fire risk by using jet ventilation and high‐flow ventilation techniques with different wattage and FiO_2_ conditions. Current literature often focuses on ETT‐related fire events, leaving investigations with varying FiO_2_ levels with high‐flow and jet ventilation underexplored. This study aims to explore the role of FiO_2_ levels and laser parameters in airway fire risk, to outline safe operating zones in oxygen‐rich environments, and to build a model that calculates excess risk as FiO_2_ levels and wattage increase.

## 2 Materials and Methods

We investigated fire risk by varying FiO_2_ and CO_2_ laser settings by using both high‐flow and jet ventilation techniques with a porcine laryngotracheal lung model in a controlled lab environment. Institutional Review Board approval was unnecessary, as human subjects were not involved.

### 2.1 Instrumentation and Surgical Equipment

The experiment utilized the MultiPulse PRO DUO system, a 10,600 nm CO_2_ laser (JenaSurgical GmbH by Asclepion Laser Technologies GmbH, Jena, Germany), equipped with an EasySpot Hybrid micromanipulator, targeting a porcine laryngeal model. The laser was paired with a Leica M320 microscope (Leica Microsystems, Wetzlar, Germany). Two ventilation techniques were applied: Infraglottic jet ventilation using an O_2_‐air mixer with a 960 Servo ventilator (Siemens‐Elema, Sweden) and Manujet III (VBM Medizintechnik GmbH, Sulz, Germany), and high‐flow ventilation using a High Flow Oxygen Blender (Sensormedics, Yorba Linda, CA, USA) connected to a Maxtec Flow Meter (Salt Lake City, UT, USA). Fume evacuation was managed by the Neptune 3 Rover (Stryker, Kalamazoo, MI, USA).

### 2.2 Experiment Setup

**FIGURE 1:**
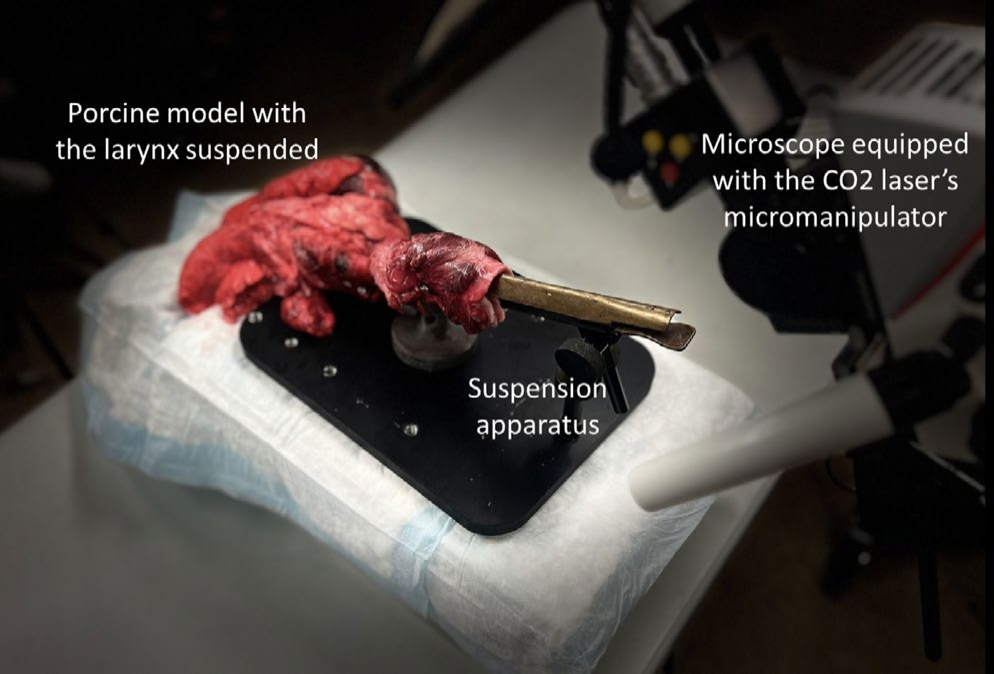
The controlled laboratory setup used for conducting the laser trials. The porcine model was secured in a suspension apparatus, while the laser was directed through the laryngoscope using a micromanipulator.

The tissue was held in place using a suspension apparatus [16] throughout the procedure (Figure 1), with video captured on a smartphone. Fire safety equipment was employed, including protective surgical eyewear, warning signs on the outside of the experimental suite, and an easily accessible fire extinguisher. In cases of an obvious fire, the oxygen supply was cut off instantly.

### 2.3 Settings and Parameters

The laser used continuous mode (CW) with no scan function for 30‐s intervals, at power levels of 4, 6, 8, 10, and 30 W. Testing the 30 W upper limit provided data on extreme flame responses, enhancing the model's ability to forecast high‐risk scenarios, even with infrequently used wattages. We systematically tested FiO_2_ levels of 50%, 70%, and 100% in combination with every power level for both ventilation techniques. Ablation was used throughout the experiment instead of cutting, as it produces more char and creates harsher conditions, allowing for a more generalized risk assessment under worst‐case scenarios.

### 2.4 Outcome Measures

**FIGURE 2:**
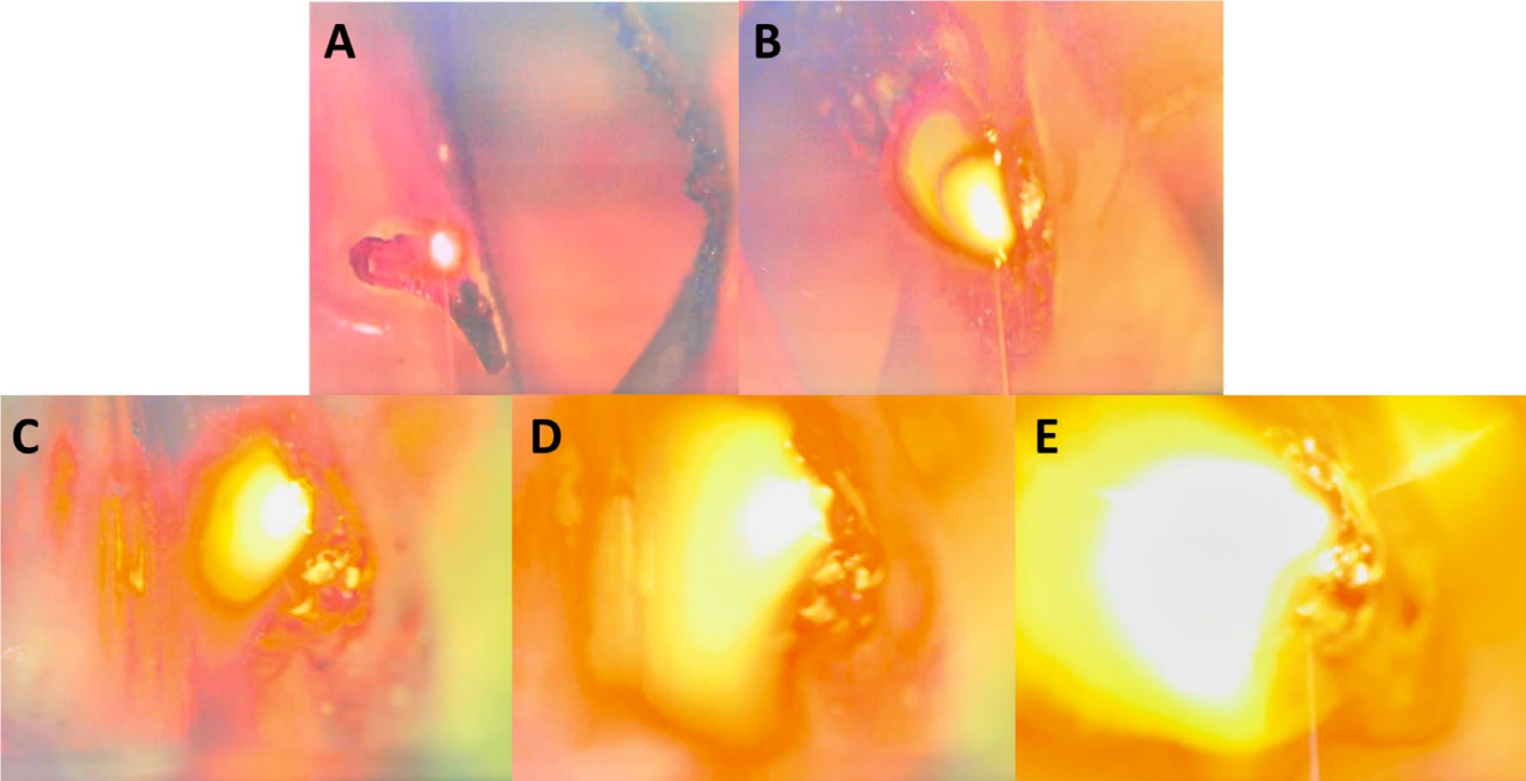
Images representing brief, expansive, and blowtorch events. (A) Brief flame: Small, transient, and confined. (B) Expansive flame: Larger, more persistent, and spreads wider. (C–E) Blowtorch effect over time, intensifying with oxygen flow.

Measurements included the time (seconds) to flame occurrence, classified as brief, expansive, or blowtorch, reflecting a spectrum of potential clinical significance. Brief flames were smaller, short‐lived, and localized, while expansive flames were larger, more prolonged, and spread wider. Blowtorch was characterized as a sustained flame, intensified with the oxygen flow. Figure 2 provides visual examples of each flame type. Expansive and blowtorch flames were identified as the most hazardous and should be entirely avoided, unlike brief flames, which pose less clinical risk. Event timing was recorded by reviewing the timer on the video footage after the experiment. In every trial, the number of both brief and expansive flames was recorded by a single reviewer (A.R.); these ratings were conducted independently of the trial conditions.

Smoke was excluded as an outcome measure because in the vast majority of trials it was not visible due to the gas flow or suction. Sparks were also excluded as an outcome measure as they were consistently seen and therefore not helpful.

### 2.5 Data Collection and Analysis

Each ventilation technique was assessed at FiO_2_ levels of 100%, followed by 70%, and then 50%. FiO_2_ levels below 50% were not studied as current literature provides sufficient evidence, with standard guidelines suggesting maintaining FiO_2_ under 30 to minimize fire risk. Before applying the laser, multiple activations of the jet or high‐flow ventilation were performed to ensure the target FiO_2_ was reached. FiO_2_ was sampled directly from the airway using a probe to verify accuracy with the CARESCAPE B450 Monitor (GE HealthCare, Chicago, Illinois, USA).

To ensure reproducibility of the experimental set up and conditions, each FiO_2_ and power parameter combination was tested at least three times, with an individual trial duration of 30 s. The laser energy was applied for the full duration unless uncontrolled fire occurred, in which case both the laser and oxygen supply were immediately shut off. When no flames appeared, the 30‐s period was documented.

### 2.6 Statistical Analysis

T‐tests were used to compare outcome measures between ventilation techniques, reported as mean ± SD. Univariate and multivariable general linear models (GLM) were employed to assess the influence of wattage and FiO_2_ level on timing and number of flames. Univariate and multivariable logistic regression models were performed to assess the relationship of FiO_2_ level and wattage with expansive flame occurrence, using a binary outcome of no expansive flame versus any detectable expansive flame. We used Julius, an AI‐based tool, to optimize the boundary delineation in the safe zone figure described below. Statistically significant differences were determined with a *p*‐value of < 0.05. The analysis was carried out using SAS version 9.4 (SAS Institute Inc., Cary, NC).

## 3 Results

A comparison of the delivered FiO_2_ and the values sampled from the airway for the tested ventilation techniques is outlined in Table 1.

**TABLE 1:** A comparison of the delivered FiO_2_ and the airway‐sampled FiO_2_ across both tested ventilation techniques.

Delivered FiO_2_ (%)	Sampled FiO_2_ (%)
100	97–99
70	69–70
50	49–50

**FIGURE 3:**
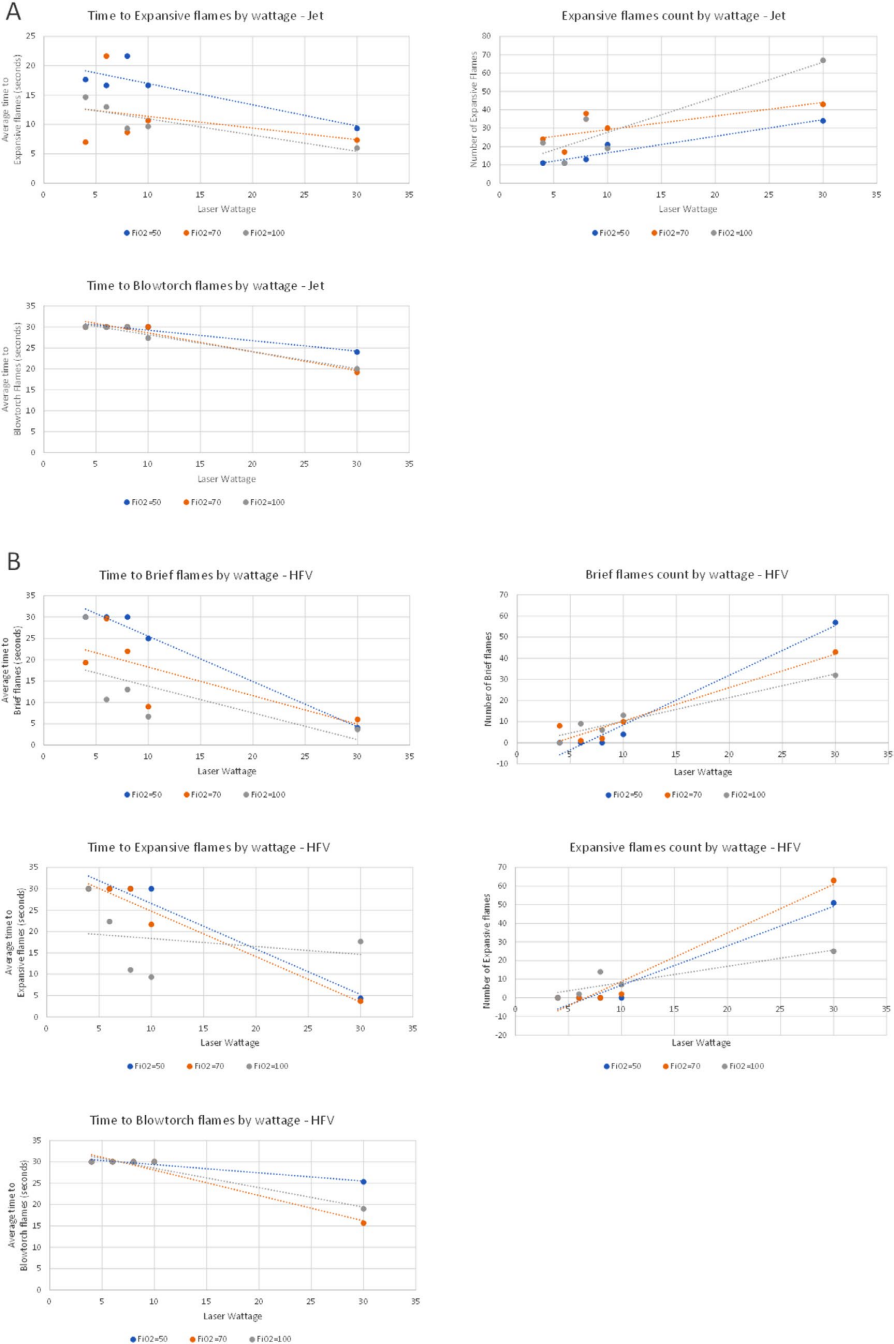
Multivariable general linear models for FiO_2_ and wattage effects for Jet (panel A) and High‐flow (panel B) ventilations. All graphs demonstrate statistical significance for wattage (*p* < 0.05).

A total of 45 jet ventilation trials were conducted, and flames appeared in every trial. Across all trials, the average time to brief flames varied between 2 and 7.6 s, while the onset of expansive flames, which ranged from 4 to 21.6 s, was longer than that of brief flames for each combination. Blowtorch effects occurred in all 30 W trials regardless of FiO_2_ levels and were also observed in the 10 W trials at 100% FiO_2_. Our general linear models show that increasing laser wattage shortens the time to expansive and blowtorch flames and increases their frequency (*p* < 0.05), as shown in Figure 3A. However, FiO_2_ increases did not significantly impact flame onset or frequency (*p* > 0.05).

**FIGURE 4:**
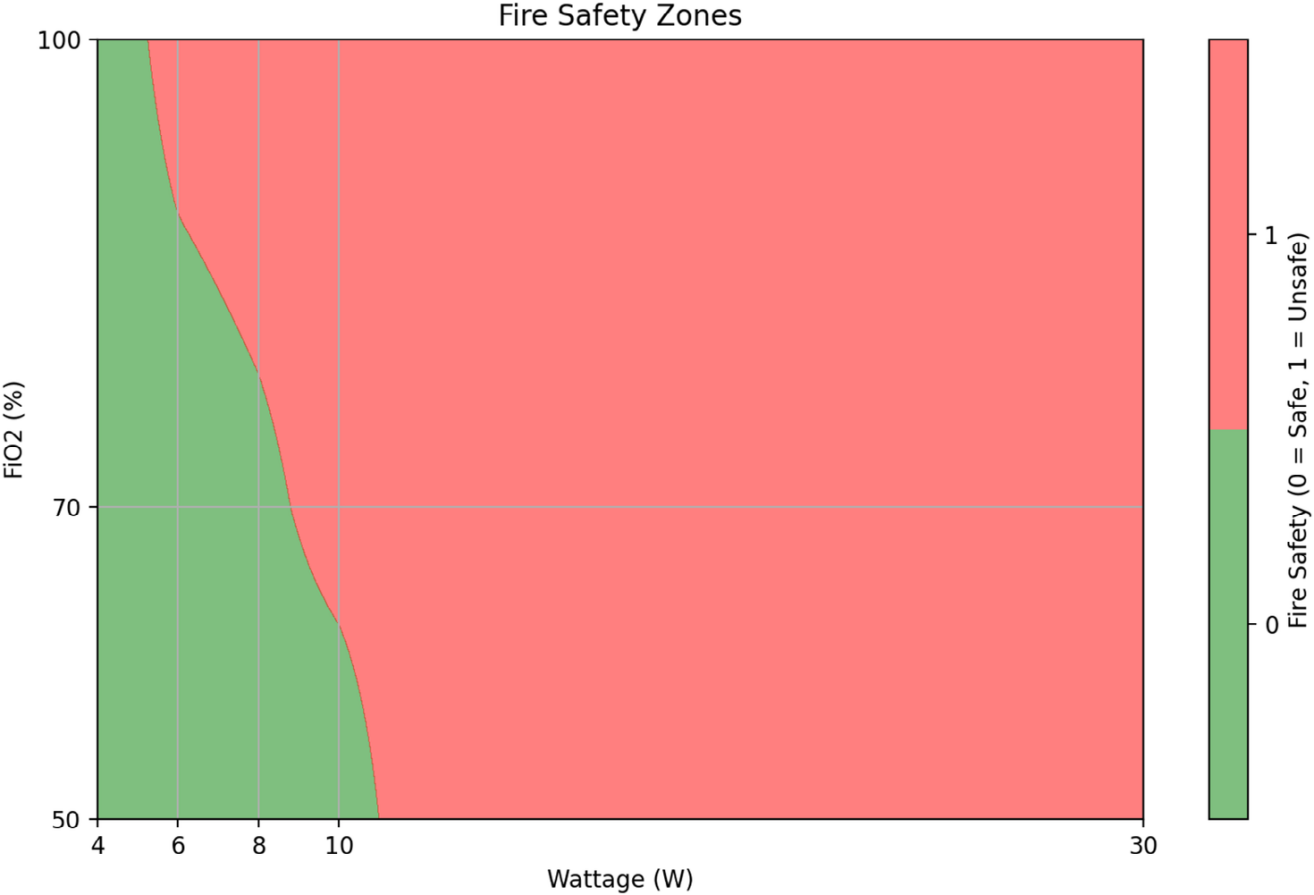
Safe zones for CO_2_ laser use in high‐flow ventilation are defined by the absence of expansive flames within 30 s at a given wattage and FiO_2_ level. These safe zones are shown in green, while red represents unsafe zones where expansive flames were observed at least once in the trials. The boundary between the zones has been optimized to create a more uniform curve. This visualization aids in identifying FiO_2_ and wattage combinations that minimize the risk of fire.

Across the 45 high‐flow ventilation trials, no expansive flames were observed at 4 W with 100%, 70%, and 50% FiO_2_, nor at 6 and 8 W with 70% and 50%, or 10 W with 50%, as illustrated in Figure 4. Of these trials, brief flames were scarcely seen at 4 W at 70%, at 6 and 8 W at 70%, and at 10 W at 50%. The time to both brief and expansive flames ranged from 3.6 to 30 s on average. Blowtorch events were present in all 30 W trials, with quicker onset as FiO_2_ increased. The multivariable GLM (Figure 3B) showed that increasing wattage significantly shortened the onset time and increased the frequency of all flame types (*p* < 0.01). In contrast, FiO_2_ only shortened the time to brief flames (*p* < 0.05) and had no effect on the time to expansive or blowtorch flames or their frequency (*p* > 0.05).

**FIGURE 5:**
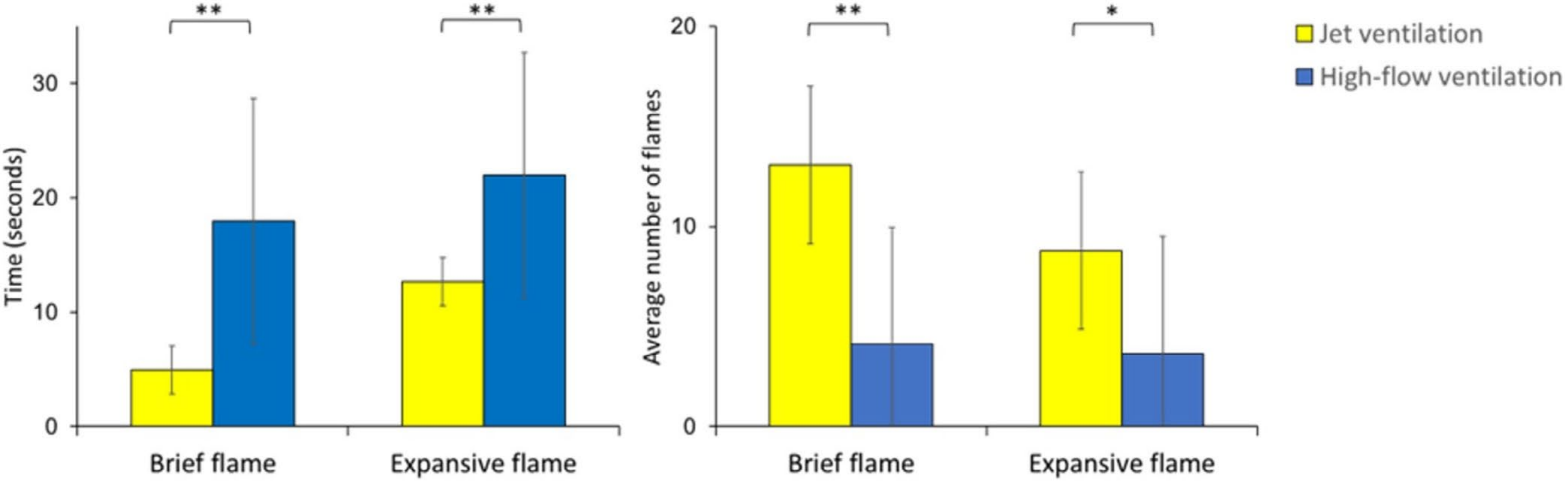
T‐tests comparing the time (seconds) to brief and expansive flames, as well as the average number of brief and expansive flames, between the Jet and High‐flow ventilation groups. Error bars represent standard errors; large error bars reflect the wide range of outcomes observed under some experimental conditions. Significant differences are denoted with an asterisk (*) for *p* < 0.05 and a double asterisk (**) for *p* < 0.01.

Comparing the two ventilation techniques, jet ventilation had shorter times to brief and expansive flames and a higher total number of flames (*p* < 0.05), as illustrated in Figure 5.

Univariate logistic regression models for FiO_2_ and laser wattage were not significant predictors of expansive flames, whereas multivariable logistic regression analysis showed a potential combined effect. However, a quasi‐complete separation warning suggests caution in interpretation.

Though not quantified, it was consistently observed that charred tissue resulted in more significant flames. On several occasions, loose charred debris was briefly engulfed by flames when hit by the laser. Another notable, though unmeasured, observation was that expansive flames in the 30 W jet trials were more substantial, dispersed, and prolonged.

## 4 Discussion

Endotracheal tubes have traditionally been utilized in airway laser surgery, yet the development of laser‐safe ETTs and improved safety protocols have helped mitigate fire risk. The greatest risk in oxygen‐enriched environments during laser surgery with laser‐safe ETT use is accidental rupture of the cuff, which may result in oxygen flooding the upper airway where the laser is applied, greatly increasing the risk of fire. To address this risk, current protocols advise lowering FiO_2_ as far as safely manageable, with 30% serving as a generally accepted threshold [11, 12, 13]. However, traditional safety recommendations may still reflect outdated practices as other ventilation techniques in airway surgery have advanced considerably as well as laser systems. To reinforce the latter, our recent experiments with the novel TruBlue laser demonstrated that higher oxygen concentrations can be safely employed with different ventilation techniques [15]. The 30% FiO_2_ limit commonly cited in the literature largely stems from several earlier studies using endotracheal tubes, with the oldest to our knowledge dating back to 1986, which identified oxygen concentration as a critical factor in airway fires. The authors stated that the risk of fire increases substantially when oxygen levels exceed 30% [8]. In our study, we aimed to determine the practical safe laser power and FiO_2_ parameters for the use of the CO_2_ laser with high‐flow and jet ventilation.

Jet ventilation studies indicate minimal to no complications in CO_2_ laser surgery, as seen in Scamman et al. [17], who reported no laser‐related complications in CO_2_ laser surgery on 10 pediatric patients. Initially, inspired gas was 100% oxygen, which was later decreased to 70% nitrous oxide in oxygen (i.e., 30% oxygen). A similar low FiO_2_ setting of 0.3–0.4 was employed by Lanzenberger‐Schragl et al. [18] in 150 CO_2_ laser cases. The Venturi effect diluted the oxygen concentration in the surgical field, and no laser‐induced complications were encountered. Aloy et al. [19] investigated a FiO_2_ of 0.4 in a cohort of over 60 patients and found no ignition‐related complications. Some studies, including 250 CO_2_ laser cases by Ruder et al. [20], didn't report FiO_2_, and no ignition incidents were noted while in Rontal et al.'s [21] review of 318 cases, one instance of jet catheter ignition was recorded. In contrast, Ihra et al. [22] examined a broad FiO_2_ range in 45 cases with combined low‐frequency and a high frequency supraglottic jet stream, starting at 1.0 and gradually lowering it to a mean of 0.6 (range 0.4–1.0). No laser or HFV complications were reported. The study indicated that FiO_2_ was in fact lower than delivered as it was diluted by surrounding air, with mean oxygen levels of 32%, 40%, and 52% at FiO_2_ values of 0.5, 0.7, and 1.0. Despite reaching higher FiO_2_ levels than previous studies, laser parameters were not explored as in our study, nor any practical recommendations were offered. To explore safety parameters, Stuermer et al. [23] aimed a laser to fat, muscle, and cartilage inside a plexiglass tube using 2–8 W under five oxygen concentrations: 21%, 30%, 40%, 50%, 70%, and 95%, simulating jet ventilation. It was concluded that lasering in oxygen concentrations greater than 50% significantly increased the risk of airway fires within 5 s, especially at 6 and 8 W. Conversely, in concentrations at or below 50%, a 5‐s laser application was considered safer, assuming sufficient smoke exhaustion. These findings largely align with our observations; however, in our porcine laryngotracheal lung model, brief flames were seen in less than 5 s at 4 W, and expansive flames, which we consider more hazardous, appear after 10 s in average. While the literature suggests CO_2_ lasers are safe at lower FiO_2_ levels, our study highlights a fire hazard with jet ventilation when the FiO_2_ exceeds 50%, even after just a few seconds of laser use at wattages as low as 4 W.

Conversely, this conclusion may differ when HFV is employed. Baudouin et al. [3] evaluated fire hazards by modifying FiO_2_, laser energy, and equipment positioning in a porcine laryngeal model for TLM. The study outcomes were dense smoke, sparks, and an airway fire. Fires only occurred when the laser targeted dry pledgets or plastic tubing. Without these materials, no fires were seen in the nine trials performed, though dense smoke developed at 5 W under FiO_2_ levels of 21% and 30%, and at 3 and 5 W with 100% FiO_2_. We opted to focus on airway clear of any extrinsic fuel and not to include pledgets in our experiment, as they were originally intended to function as a heat sink and mechanical barrier to prevent cuff rupture by absorbing the laser. In HFV, there is no cuff to avoid, and pledgets used for hemostasis are not used with active laser use [24]. Moreover, for vocal fold protection, dedicated instruments are more appropriate. Baudouin found that HFV with up to 5 W laser energy and 100% FiO_2_ carried a very low risk of fire, owing to the lack of combustible materials. Similarly, in our experiment, we concluded that 4 W at 100% FiO_2_ is safe under the tested conditions. Nevertheless, we found higher wattages are potentially safe with reduced FiO_2_ levels, as outlined in Figure 4.

In a prospective clinical study by Novakovic et al. [25], oxygen concentration was reduced from 100% to 30% before the laser procedure, then raised it to 100% after the procedure. This method provided a safe lasering window of 4.5 min, with no fires or other significant complications. Similarly, no airway fires or adverse events were reported in a small retrospective cohort of 21 patients undergoing CO_2_ laser procedures with HFV [26]. Though laser parameters were not detailed in either study, they were better described in a larger retrospective cohort by Khan et al. [27], who evaluated 136 CO_2_ laser procedures performed using Transnasal Humidified Rapid‐Insufflation Ventilatory Exchange (THRIVE). No fires occurred when strict guidelines were followed, including limiting CO_2_ laser power to less than 5–6 W for micromanipulator‐guided or below 10–12 W for fiber‐based CO_2_ laser, ensuring laser exposure did not exceed 5 s, and ensuring no flammable materials were present in the airway. Our study aligned with these conclusions, with one distinction: we demonstrated that continuous laser application for 30 s at 4 W with 100% FiO_2_ resulted in neither brief nor expansive flames.

Our study also compared fire risk between jet ventilation and high‐flow ventilation, showing HFV is significantly safer in terms of time to ignition time and the number of flames. Furthermore, we noticed that jet ventilation tends to increase flame intensity more than HFV, and charred tissue appears to be more susceptible to ignition under jet ventilation. This latter finding underlines that intrinsic materials like native tissue can also cause ignition in certain scenarios, challenging the common assumption that fuel sources are necessarily extrinsic (e.g., pledgets, tubes). Charred tissue, serving as intrinsic fuel, appears to markedly increase flame likelihood, leading to a vicious cycle of more char and more fire. Therefore, charred tissue should be meticulously addressed in oxygen‐rich laser surgery with jet and high‐flow ventilation.

We calculated the excess risk associated with wattage increases using general linear models (Figure 3). Intriguingly, wattage increase was statistically significant, while FiO_2_ changes showed less robust statistical significance. This finding challenges the assumption that FiO_2_ level is the primary factor in fire risk, highlighting instead that wattage may play a more critical role in fire safety in oxygen‐rich environments. Having said that, the quasi‐complete separation observed in the multivariable logistic regression model suggests that both FiO_2_ and wattage may reliably predict expansive flame risk, indicating their potential use in establishing safety thresholds. However, this separation limits statistical modeling, restricting the ability to fully quantify the independent influence of each factor (FiO_2_ or wattage) on flame risk. Further studies with finer FiO_2_ and wattage increments, and larger sample sizes may help to refine these findings. Furthermore, FiO_2_ levels below 50% were not included in this study. Although current guidelines support the use of FiO_2_ < 30% for safety, empirical validation within this model would strengthen confidence in its generalizability and rule out any experimental bias.

To further address our study's limitations, we opted for ablation over excision, as ablation generates more char, creating conditions more likely to generate fire. While these conditions may not fully replicate the fire risks during excision, we believe testing under worst‐case scenarios allows for a more generalized risk assessment. If excision had been chosen, the conclusions might have been less alarming, which could provide a false sense of security. Further studies focusing on charring as an independent variable could better clarify its role as a risk factor.

Additionally, while the cadaver larynx, trachea, and lung model provides insights into human airway responses, it falls short of replicating exact physiological conditions, notably in oxygen transport along the airways. To address this, we included FiO_2_ levels up to 100% for a comprehensive assessment. Since we recorded single‐time FiO_2_ levels, fluctuations during prolonged procedures may have impacted results. Continuous monitoring could better capture these variations but requires a foreign sampling line in the airway during laser use. Moreover, omitting humidification in HFV trials led to drier airways, likely elevating fire risk; this setup reinforces the real‐world applicability of our results under harsher conditions. Though aiming for real‐world applicability, the absence of multiple raters and formal inter‐rater assessment reduced objectivity; however, the grading was performed independently and focused on clinical relevance, thereby retaining its practical value.

## 5 Conclusion

Our porcine model experiment demonstrates safe zones for CO_2_ laser treatment with high‐flow ventilation: 4 W at 100% FiO_2_, 6 and 8 W at 70% FiO_2_, and 10 W at 50% FiO_2_—settings where no expansive flames were observed. This is provided the laser application does not exceed 30 s, pending translation to clinical environments. Notably, no flames of any kind occurred at 4 W at 100% and 50% FiO_2_, 6 and 8 W at 50% FiO_2_. CO_2_ laser application with high‐flow ventilation was found to be significantly safer than with jet ventilation, as expansive flame events occurred in all jet ventilation trials, even at 4 W with FiO_2_ levels as low as 50%. Based on these findings, we recommend maintaining FiO_2_ at 30% for jet ventilation laser procedures to minimize the risk of hazardous flames. We also identified laser wattage as a key factor in reducing airway fire risk during laser surgery, with FiO_2_ levels playing a secondary role. However, refining these results in future studies with finer increments of FiO_2_ and wattage, along with larger sample sizes, may be beneficial. Through the calculation of excess risk from wattage increments, our multivariable model provides surgeons with the data needed to assess fire risk more effectively, based on the laser parameters and FiO_2_ settings selected for their procedures. Our results highlight the critical role of ventilation choice and laser settings in reducing fire hazards, thereby enhancing patient safety during laser surgery.

## Disclosure

The authors have nothing to report.

## Conflicts of Interest

The authors declare no conflicts of interest.
